# Curcumin-Induced Apoptosis in Human Hepatocellular Carcinoma J5 Cells: Critical Role of Ca^+2^-Dependent Pathway

**DOI:** 10.1155/2012/512907

**Published:** 2012-04-01

**Authors:** Wei-Hsun Wang, I-Tsang Chiang, Kuke Ding, Jing-Gung Chung, Wuu-Jyh Lin, Song-Shei Lin, Jeng-Jong Hwang

**Affiliations:** ^1^Department of Biomedical Imaging and Radiological Sciences, National Yang-Ming University, Taipei 112, Taiwan; ^2^Department of Orthopedic Surgery, Changhua Christian Hospital, Changhua 500, Taiwan; ^3^National Institute for Radiological Protection, Chinese Center for Disease Control and Prevention, Beijing 100088, China; ^4^Department of Biological Science and Technology, China Medical University, Taichung 404, Taiwan; ^5^Division of Radioisotope, Institute of Nuclear Energy Research, Taoyuan 325, Taiwan; ^6^Department of Radiological Technology, Central Taiwan University of Science and Technology, Taichung 406, Taiwan

## Abstract

The antitumor effects of curcumin, a natural biologically active compound extracted from rhizomes of Curcuma longa, have been studied in many cancer cell types including human hepatocellular carcinoma (HCC). Here, we investigated the effects of Ca^2+^ on curcumin-induced apoptosis in human HCC J5 cells. The abrogation of mitochondrial membrane potential (ΔΨ_m_), the increase of reactive oxygen species (ROS) production, and calcium release were demonstrated with flow cytometry as early as 15 minutes after curcumin treatment. In addition, an increase level of cytochrome c in the cytoplasm which led to DNA fragmentation was observed. To verify the role of Ca^2+^ in curcumin-induced apoptosis, 1,2-bis(o-aminophenoxy)ethane-N,N,N′,N′-tetraacetic acid (BAPTA), an intracellular calcium chelator, was applied. Cell viability was increased, but ΔΨ_m_, ROS production, activation of caspase 3, and cell death were decreased in J5 cells pretreated with BAPTA for 2 h followed by the treatment of 25 *μ*M curcumin. These results suggest that the curcumin-induced apoptosis in human HCC J5 cells is via mitochondria-dependent pathway and is closely related to the level of intracellular accumulation of calcium.

## 1. Introduction

Human HCC treated with chemotherapy often turned out with poor prognosis [1, 2]. Curcumin, one of phytochemical compounds, has been shown with chemopreventive and chemotherapeutic properties against tumors in animal models and clinical trials [3–5]. Curcumin induces the apoptosis of tumor cells through mitochondria-dependant pathways, including the release of cytochrome c, changes in electron transport, and loss of mitochondrial transmembrane potential [6]. Curcumin can stimulate intracellular Ca^2+^ uptake into the mitochondria [7], resulting in the stimulation of oxidative phosphorylation, transmission, and amplification of the apoptotic signal via the suppression of mitochondria membrane potential and the release of cytochrome c [8]. Apoptosis induced by curcumin in human HepG2 cells has been shown through mitochondrial hyperpolarization and DNA damage [9]. Mitochondria are the moderator of intracellular Ca^2+^ dynamics and transport through a complex system consisting of two modes of influx and efflux [7]. Oxidative phosphorylation can be stimulated by the accumulation of Ca^2+^ in the mitochondrial matrix, then transmit and amplify the apoptotic signal [8]. Apoptosis induced by curcumin also has been reported through the prevention of intracellular Ca^2+^ depletion and the release of cytochrome c in mouse melanoma cells [10]. We hypothesize that curcumin-induced Ca^2+^ release will result in mitochondrial Ca^2+^ overuptake to affect mitochondria membrane potential stability. To prove this, we choose BAPTA, an intracellular Ca^2+^ chelator, as the inhibitor for mitochondrial Ca^2+^ uptake [11]. However, previous study indicates that curcumin-induced apoptosis is through ER stress dependent pathway, that is, GADD153 transcription activation [12]. In this study, we demonstrated that curcumin-induced apoptosis in human HCC J5 cells is via Ca^2+^-regulated mitochondria-dependent pathway.

## 2. Materials and Methods

### 2.1. Cell Culture

The HCC J5 cell line was obtained from the Cell Culture Center of the National Taiwan University (Taipei, Taiwan). Cells were cultured with DMEM supplemented with 2 mM L-glutamine, 1.5 g/L sodium bicarbonate, 10% fetal bovine serum, and 2% penicillin-streptomycin (10,000 U/mL penicillin and 10 mg/mL streptomycin in a 5% CO_2_ humidified incubator).

### 2.2. Morphological Study and Cell Viability

The J5 cells were cultured in 12-well plates at a density of 2 × 10^5^ cells/well for 24 h, then treated with various concentrations of curcumin (0, 10, 15, 20, 25, and 50 *μ*M in 0.1% DMSO) for different time periods. Trypan blue exclusion was used to the cell viability as previously described [13]. In short, approximately 10 *μ*L of cell suspensions in PBS were mixed with 40 *μ*L of trypan blue. The numbers of stained (dead cells) and unstained cells (live cells) were counted under a light microscope. At least, 5000 cells were counted. The cell viability is calculated using the following formula:

(1) $$\text{Cell viability = }\frac{\text{unstained cells}}{\text{unstained cells + stained cells }}\times 100\%.$$

### 2.3. Comet Assay

2 × 10^5^ J5 cells/well were grown in 12-well plates and treated with curcumin at 0, 25, and 50 *μ*M for 24 h, then examined for DNA damage using Comet assay. Cells were harvested and mixed with low melting point agarose. The mixture was then placed in the solid normal melting point agarose on the slide covered with coverslip. The coverslip was removed after the agarose was gelled at 4°C. The slide was transferred to the lysis buffer at 4°C for 1 h before putting in alkaline buffer for electrophoresis (25 V, 300 mA). The slide was washed with neutralized buffer and stained with PI after electrophoresis [13].

### 2.4. DNA Fragmentation

1 × 10^6^ J5 cells/well were grown in 6-well plates and treated with 25 *μ*M curcumin for 12, 24, 36, and 48 h. The fragmented DNA was extracted using a cell genomic DNA purification kit (Genemark). The DNA extracted procedures followed the protocols provided by the manufacture. The DNA fragmentation was assayed with 1.5% agarose gel electrophoresis.

### 2.5. Caspase-3 Activity Assay

2 × 10^5^ J5 cells/well were cultured in 12-well plates and treated with 25 *μ*M curcumin for various time periods. Cells were harvested in a 15-mL centrifuge tube by centrifugation. 50 *μ*L of 10 *μ*M PhiPhilux solution, a substrate for caspase-3, was added to each well and incubated at 37°C for 1 h. Cells were then washed once with 1 mL of ice-cold PBS and resuspended in fresh 1 mL PBS. Caspase-3 activity was analyzed by flow cytometry (Becton-Dickinson, CA, USA) equipped with an argon ion laser at 488 nm wavelength [13]. In addition, J5 cells were pretreated with 10 *μ*M 1,2-bis(o-aminophenoxy)ethane-N,N,N′,N′-tetraacetic acid (BAPTA), a calcium chelator, for 2 h, then were assayed for caspase-3 activity as described in the above.

### 2.6. Detection of Reactive Oxygen Species (ROS)

2 × 10^5^ J5 cells/well in 12-well plates were incubated with 25 *μ*M curcumin for different time periods to detect the changes of ROS. Cells were harvested and washed twice, resuspended in 500 *μ*L of 10 *μ*M 2,7-dichlorodihydrofluorescein diacetate (DCFH-DA), and incubated at 37°C for 30 min, then analyzed by flow cytometry [13].

### 2.7. Detection of Mitochondrial Membrane Potential (ΔΨ_m_)

2 × 10^5^ J5 cells/well in 12-well plates were incubated with 25 *μ*M curcumin for different time course to determine the changes in ΔΨ_m_. Cells were harvested and washed twice, resuspended in 500 *μ*L of 4 *μ*M DiOC_6_, and incubated at 37°C for 30 min, then analyzed by flow cytometry [13].

### 2.8. Cell Viability, ROS Production, ΔΨ_m_ Levels in J5 cells Pre-Treated with BAPTA

2 × 10^5^ J5 cells/well in 12-well plates were pre-treated with 100 *μ*M BAPTA for 2 h, then treated with 25 *μ*M curcumin for 24 h. Cells were harvested and washed twice, half of cells were analyzed for cell viability with PI staining, the rest was resuspended in 4 *μ*M DiOC_6_ and 10 *μ*M DCFH-DA before incubated at 37°C for 30 min, then analyzed by flow cytometry.

### 2.9. Determination of Ca^2+^ Concentration

2 × 10^5^ J5 cells/well in 12-well plates were incubated with 25 *μ*M curcumin for various time intervals to determine the Ca^2+^ levels. Cells were harvested and washed twice, resuspended in 3 *μ*g/mL Indo 1/AM, incubated at 37°C for 30 min, and analyzed by flow cytometry.

### 2.10. Western Blotting

2 × 10^5^ J5 cells/well in 12-well plates were treated with 25 *μ*M curcumin for 0, 6, 12, 24, and 48 h. The level of cytochrome c in the cytosol was isolated according to the manufacturer's protocol (A cytosol/nuclear extraction kit purchased from Chemicon International, Temecula, CA, USA). The total proteins of cells were extracted with cell lysis buffer (50 mM Tris-HCL pH 8.0, 120 mM NaCl, 0.5% NP-40, 1 mM PMSF), and 40 *μ*g of protein extract was separated by 10% SDS-PAGE, then transferred to a polyvinylidene difluoride (PVDF) membrane (Bio-Rad), blocked with 5% nonfat milk in TBSTween buffer (0.12 M Tris-base, 1.5 M NaCl, 0.1% Tween20) for 1 hour at room temperature, and incubated with the appropriate antibody overnight at 4°C, then incubated with horseradish peroxidase conjugated secondary antibody for 30 min at room temperature. The bound antibody was detected with peroxidase-conjugated anti-rabbit antibody (1 : 10000) or anti-mouse antibody (1 : 10000) followed by chemiluminescence (ECL System) and exposed by autoradiography. The following primary antibodies except cytochrome c (1 : 500) (Oncogene Research Products): *β*-actin (1 : 10000), Bcl-2 (1 : 1000), Bcl-xl (1 : 1000), Fas (1 : 1000), caspase-8 (1 : 1000), caspase-12 (1 : 1000), and catalase (1 : 1000) were purchased from Upstate, Millipore.

### 2.11. Statistics

Student's *t*-test was used to evaluate the significance or *P* values between groups (*P < 0.05, **P < 0.01). Standard errors of mean values were depicted as error bars in all figures.

## 3. Results

### 3.1. Morphological Study and Cell Viability

**Figure 1:**
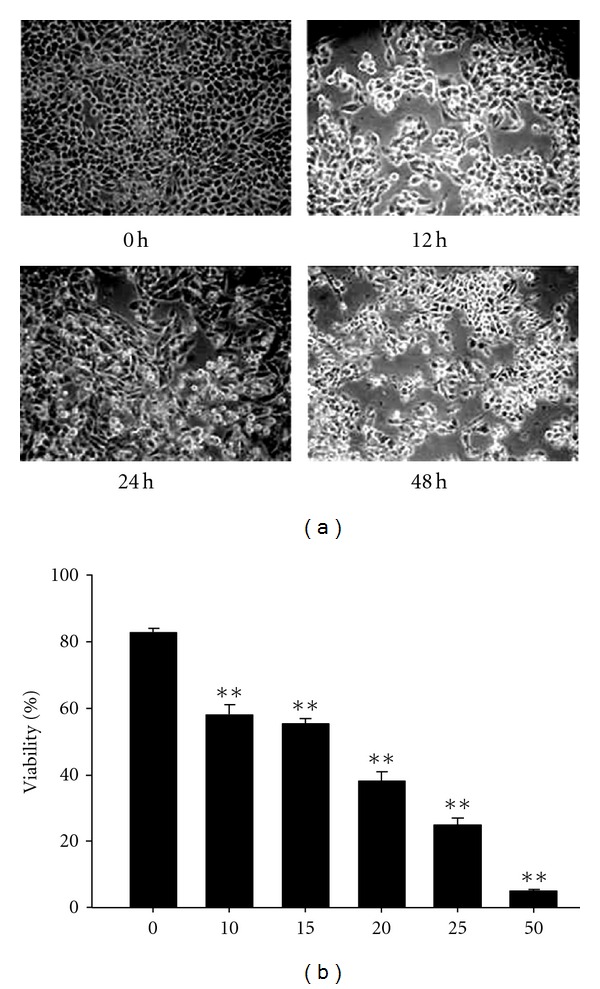
The cell survival of J5 cells after curcumin treatment. J5 cells were treated with 25 *μ*M curcumin for 0, 12, 24, and 48 h and photographed with phase-contrast microscope (a) or with various doses of curcumin for 24 h, and cell viability was determined by trypan blue exclusion assay (b). Each data point is the mean ± SE of three experiments. **P < 0.01 stands for significant difference as compared with that of the control.

The morphology of J5 cells induced by curcumin was remained unchanged, but the apoptotic bodies could be observed (Figure 1(a)), and increased with times.  Figure 1(b) shows the viability of J5 cells are decreased with the increase of curcumin concentration (10–50 *μ*M).

### 3.2. Ca^2+^ Production, Mitochondria Membrane Potential (ΔΨ_m_), and Production of Reactive Oxygen Species (ROS) Affected by Curcumin in J5 Cells

**Figure 2:**
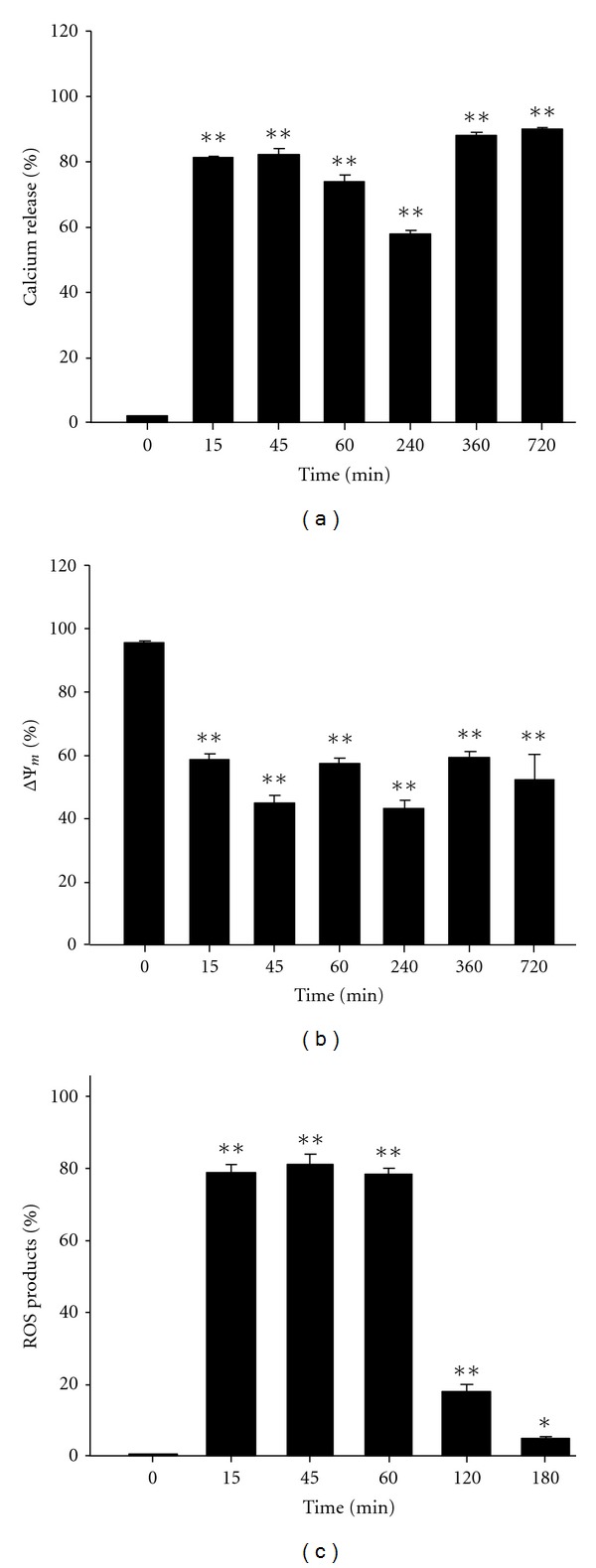
Flow cytometric analysis of Ca^2+^ concentration, mitochondrial membrane potential (ΔΨ_m_), and reactive oxygen species (ROS) in J5 cells treated with curcumin. 5 × 10^5^ J5 cells/well in 12-well plates were incubated with 25 *μ*M curcumin for different time periods. (a) The release of Ca^2+^ as a function of time. (b) Mitochondrial membrane potential (ΔΨ_m_) assay. (c) Changes of ROS production as a function of time. Each data point is the mean ± SE of three experiments. *P < 0.05  and **P < 0.01 are significantly different from that of the control.

Figure 2(a) showed that Ca^2+^ production was significantly enhanced from 15 min up to 720 min by 25 *μ*M curcumin treatment, while the mitochondria membrane potential (ΔΨ_m_) was significantly decreased (Figure 2(b)) as compared with that of the control. Reactive oxygen species (ROS) was also significantly increased and reached the highest levels at 15–60 min after 25 *μ*M curcumin treatment (Figure 2(c)).

### 3.3. The Release of Cytochrome c and Apoptotic-Associated Proteins Affected by Curcumin in J5 Cells

**Figure 3:**
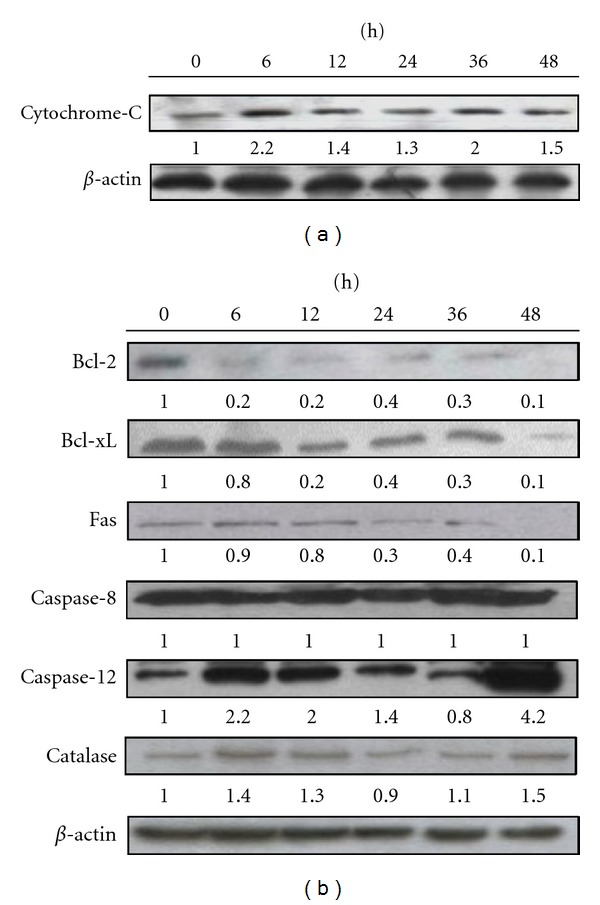
Western blot demonstrates the changes on the levels of proteins in J5 cells after treated with curcumin. J5 cells were treated with 25 *μ*M curcumin for different time periods before the cytosolic fraction and total protein were extracted from the cells. (a) The cytochrome C was increased with times. (b) Bcl-2, Bcl-xL, Fas, caspase-8, caspase-12, and catalase were assayed. *β*-actin was used as an internal control.

To characterize the molecular mechanisms of curcumin-induced apoptosis in J5 cells, the expressions of apoptotic-associated proteins were examined with Western blotting.  Figure 3(a) showed that cytochrome c was released from the mitochondria to the cytosol in J5 cells treated with 25 *μ*M curcumin for different time periods (6–48 h). On the other hand, the protein levels of Bcl-2, Bcl-xL, and Fas were decreased. Both caspase-12 and catalase were increased after curcumin treatment for 6 and 12 h, but decreased for 24 and 36 h, then increased again for 48 h. Procaspase-8 were not affected by curcumin treatment.

### 3.4. DNA Damage and Fragmentation Caused by Curcumin in J5 Cells

**Figure 4:**
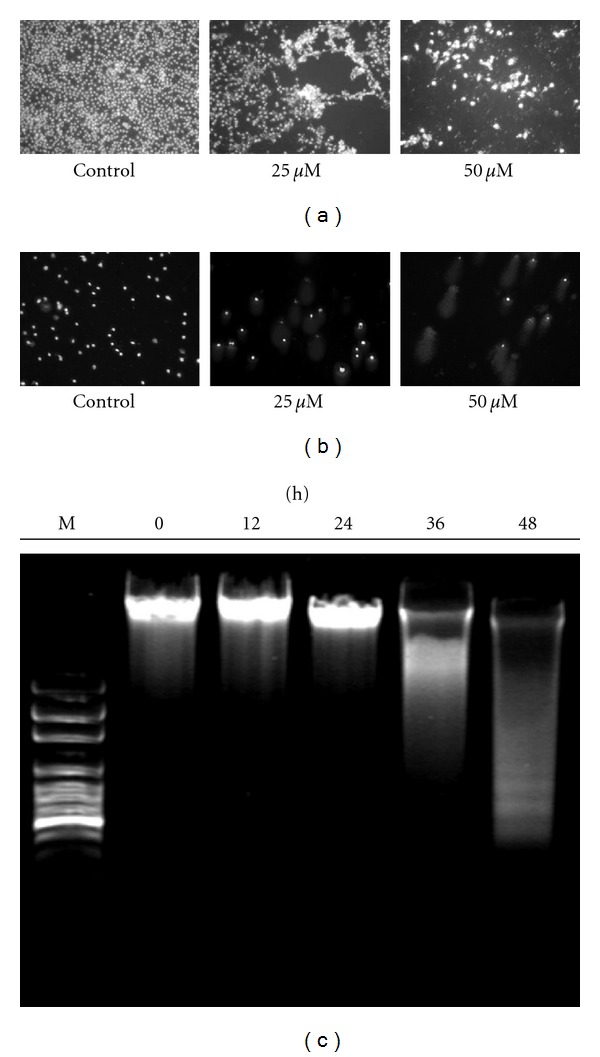
DNA damage and DNA fragmentation were induced by curcumin in J5 cells. Cells were incubated with 0, 25, and 50 *μ*M curcumin for 24 h, and DNA damage was examined by (a) DAPI staining, (b) comet assay, and photographed by fluorescence microscope. (c) J5 cells were treated with 25 *μ*M curcumin for 0, 12, 24, 36, and 48 h, and DNA fragmentation was determined with DNA gel electrophoresis.

DAPI staining was used to detect the DNA damage in J5 cells treated with curcumin.  Figure 4(a) showed that the nuclei of control cells were round and smaller as compared with the condensed and larger nuclei of cells exposed to 25 and 50 *μ*M curcumin for 24 h. The DNA damage induced by curcumin was in a dose-dependent manner. The Comet assay also showed the similar results. The 50 *μ*M curcumin treatment showed a longer DNA migration smear (Figure 4(b)), indicating that more DNA was damaged in the cells. DNA fragmentations were found in J5 cells after12, 24, 36, and 48 h of continuous exposure to 25 *μ*M curcumin as shown in Figure 4(c). The induction of DNA fragmentation by curcumin was in a time-dependent manner.

### 3.5. Effects of Calcium Chelator BAPTA on Cell Viability, ΔΨ_m_, ROS Production, and Caspase-3 Activity Induced by Curcumin in J5 Cells

**Figure 5:**
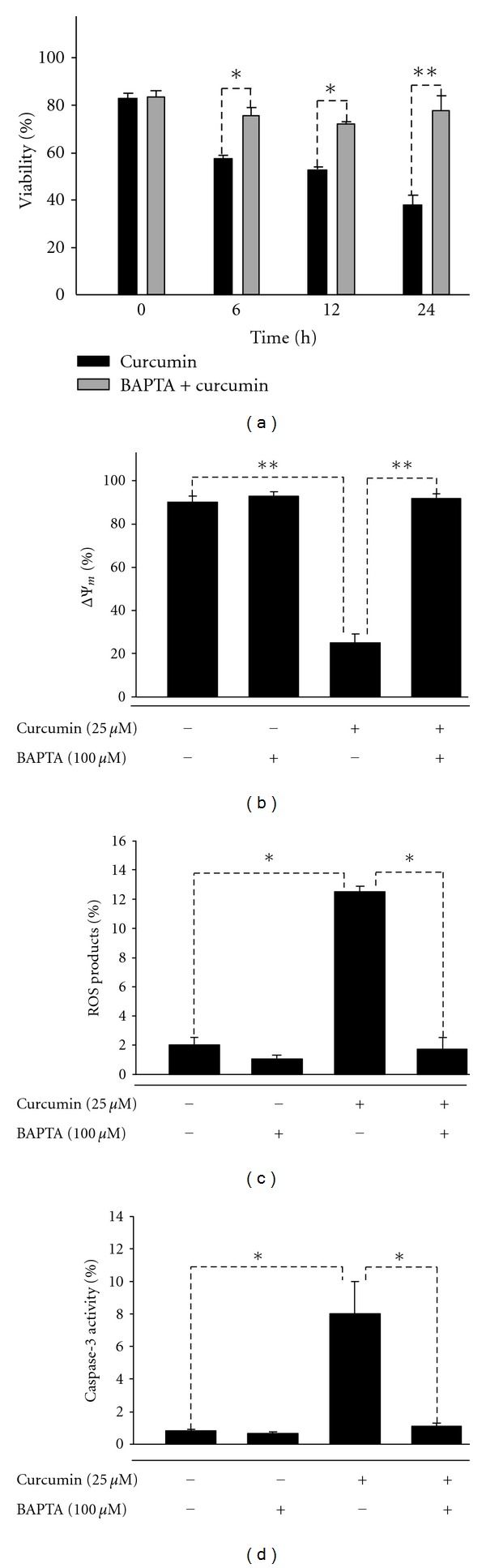
Effects of pretreated BAPTA (Ca^2+^ chelator) for 2 h followed by the treatment of 25 *μ*M curcumin in J5 cells on cell viability, ΔΨ_m_, and ROS production. (a) cell viability, (b) ΔΨ_m_, (c) ROS production, and (d) caspase-3 activity. Each data represents mean ± SE of three experiments. (*P < 0.05, **P < 0.01).

J5 cells were pretreated with 100 *μ*M BAPTA for 2 h, followed by incubation with 25 *μ*M curcumin for different time periods. Cell viability, ΔΨ_m_, ROS, and caspase-3 activity were analyzed by flow cytometry.  Figure 5(a) showed that BAPTA could rescue the cell death from curcumin treatment. The recovery of mitochondria membrane potential ΔΨ_m_ and the inhibition of ROS by BAPTA were shown in Figures 5(b) and 5(c), respectively. In addition, caspase-3 activity increased by 25 *μ*M curcumin was inhibited by BAPTA.

## 4. Discussion

We have demonstrated that DNA damage and endoplasmic reticulum (ER) stress-mediated curcumin-induced cell cycle arrest and apoptosis are through the activation of caspases, and mitochondria-dependent pathways in A549 cells [13]; here we further show the similar finding in human hepatocellular carcinoma J5 cells. Mitochondrial dysfunction associated with apoptosis is characterized with the loss of mitochondrial membrane potential (ΔΨ_m_), permeability transition, and the release of cytochrome c from the mitochondria into the cytosol [14]. We also show that curcumin induces apoptosis in human HCC J5 cells via mitochondrial-dependent pathway with the suppression of both mitochondria membrane potential (ΔΨ_m_) and the induction of cytochrome c release; nevertheless, the ROS production is induced and the Ca^2+^ in cytoplasm is accumulated. Other than mitochondrial dysfunction, the mechanisms responsible for curcumin-induced apoptosis in different cancer cell types may also involve the activation of caspases, and the inhibition of antiapoptotic Bcl-2 family proteins [15–17]. We also found that curcumin decreased the protein levels of Bcl-2 and Bcl-xL in this study. Dröge et al. reported that high levels of ROS induce DNA damage, and result in the cell death [18]. Our result indicates that ROS production in J5 cells with the highest levels at 15–60 min after 25 *μ*M curcumin treatment. Both superoxide dismutase (SOD) and catalase of ROS scavenger reduced ROS production [19]. We also found that curcumin increased protein levels of catalase after curcumin treatment for 6 and 12 h in J5 cells. ΔΨ_m_ depletion, cytochrome c release, ROS production, and DNA damage caused by curcumin all have contribution on the cell death. However, neither of the aforementioned results, in which multiple related mechanisms of curcumin-induced apoptosis was revealed, indicate the key molecule with potential to steer the pharmacologic effect of curcumin.

Intracellular-free calcium ([Ca^2+^]_i_) is a universal signaling molecule regulating many cellular functions including apoptosis. In addition, Ca^2+^-dependent processes are closely related with the mainstream apoptosis executioners, that is, caspases [20]. It is also shown to activate and modulate the execution of a nonapoptotic cell death in C. elegans [21]. Both the overload and the depletion of endoplasmic reticulum Ca^2+^ pool result in the induction of ER stress, and further initiate the apoptotic pathway via activation of procaspase-12, which is transferred to the ER membrane during ER stress in response to the mobilization of intracellular Ca^2+^ stores [20, 22]. Once activated, caspase-12 acts on the effector caspases to induce apoptosis [23]. We also found the highest protein level of caspase-12 at 48 h after curcumin treatment in this study. Furthermore, the disruption of mitochondrial membrane potential and the disturbance of intracellular free Ca^2+^ concentration were also found to be affected by curcumin [24].

In order to elucidate the mechanism that how Ca^2+^ was involved in the curcumin-induced cell death, human HCC J5 cells were pretreated with BAPTA, a calcium chelator, followed by the curcumin treatment. The result showed that BAPTA could reverse curcumin-induced cell death, despite the fact that ER stress is able to activate apoptosis [25]. Although the previous study of curcumin-induced apoptosis in HCC J5 cells displays increased ER stress hallmark GADD153 [12], our finding implicates the major significance of Ca^2+^-dependent mechanism in curcumin-induced apoptosis. A similar result has been suggested by Bae et al. in human leukemia cell line as well [7]. Notably, BAPTA also inhibited the depletion of mitochondria membrane potential, ROS production, and capase-3 activation in human HCC J5 cells. In conclusion, our results suggest that the apoptosis induced by curcumin in human HCC J5 cells is through mitochondria-dependent pathway, in which Ca^2+^ release plays an important role.
